# China's top 10 breakthroughs in science and technology in 2023

**DOI:** 10.1093/nsr/nwae084

**Published:** 2024-03-18

**Authors:** He Zhu

**Affiliations:** NSR in Beijing, China

**Figure:**
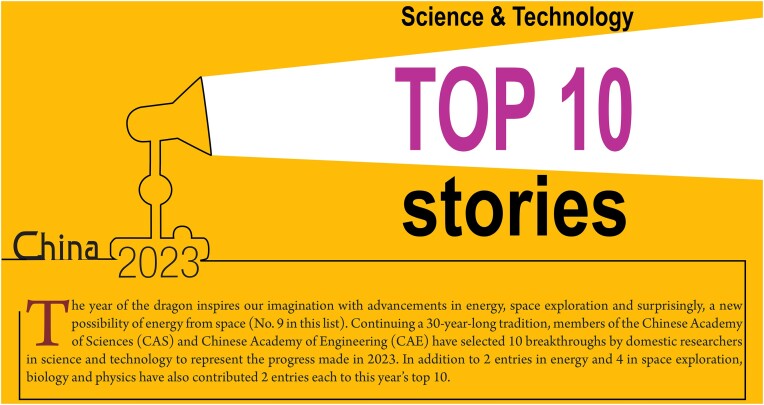


## 1. Commercial operation started at the first fourth-generation nuclear power plant in the world.

**Figure 1.**
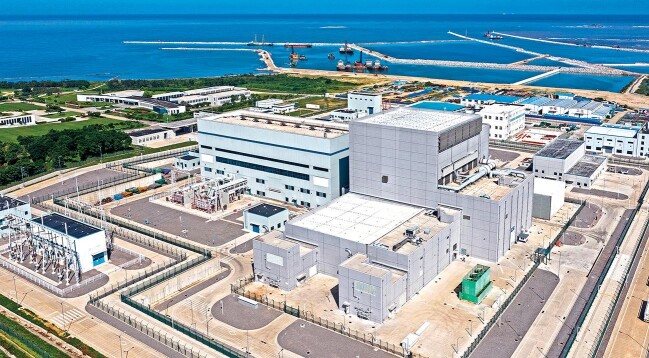
Shidaowan is the first high-temperature gas-cooled nuclear reactor in the world. (Courtesy of China Huaneng Group.)

The Shidaowan high-temperature gas-cooled reactor constructed by China Huaneng Group started commercial operation on 6 December 2023. As the first fourth-generation modular nuclear reactor, Shidaowan establishes China as a worldwide leader in high-temperature gas-cooled nuclear technology (Fig. 1). China Huaneng Group owns all intellectual properties involved in Shidaowan, making this achievement a significant step towards China's self-reliance in science and technology.

High-temperature gas-cooled reactors are recognized internationally as advanced fourth-generation nuclear reactors, representing the direction of future nuclear technology. In the event of a total loss of cooling power, this type of reactor does not need any intervention to remain safe and experiences no risk of reactor core meltdown or radioactive leak. China Huaneng Group led the development of Shidaowan in collaboration with Tsinghua University and China National Nuclear Corporation. This reactor project was approved as a national major R&D project in 2006, with construction starting in 2012. China Huaneng Group took full advantage of its supply chains in China and developed and manufactured 93.4% of the components.

## 2. Return of Shenzhou 16 after the first manned mission in the application and development stage of the China Space Station.

The return capsule of the Shenzhou 16 spacecraft successfully landed at 8:11 on 31 October 2023 at the Dongfeng Landing Site. Medical staff verified that astronauts Haipeng Jing, Yangzhu Zhu and Haichao Gui returned in good health, marking the successful completion of the manned mission of Shenzhou 16.

**Figure 2:**
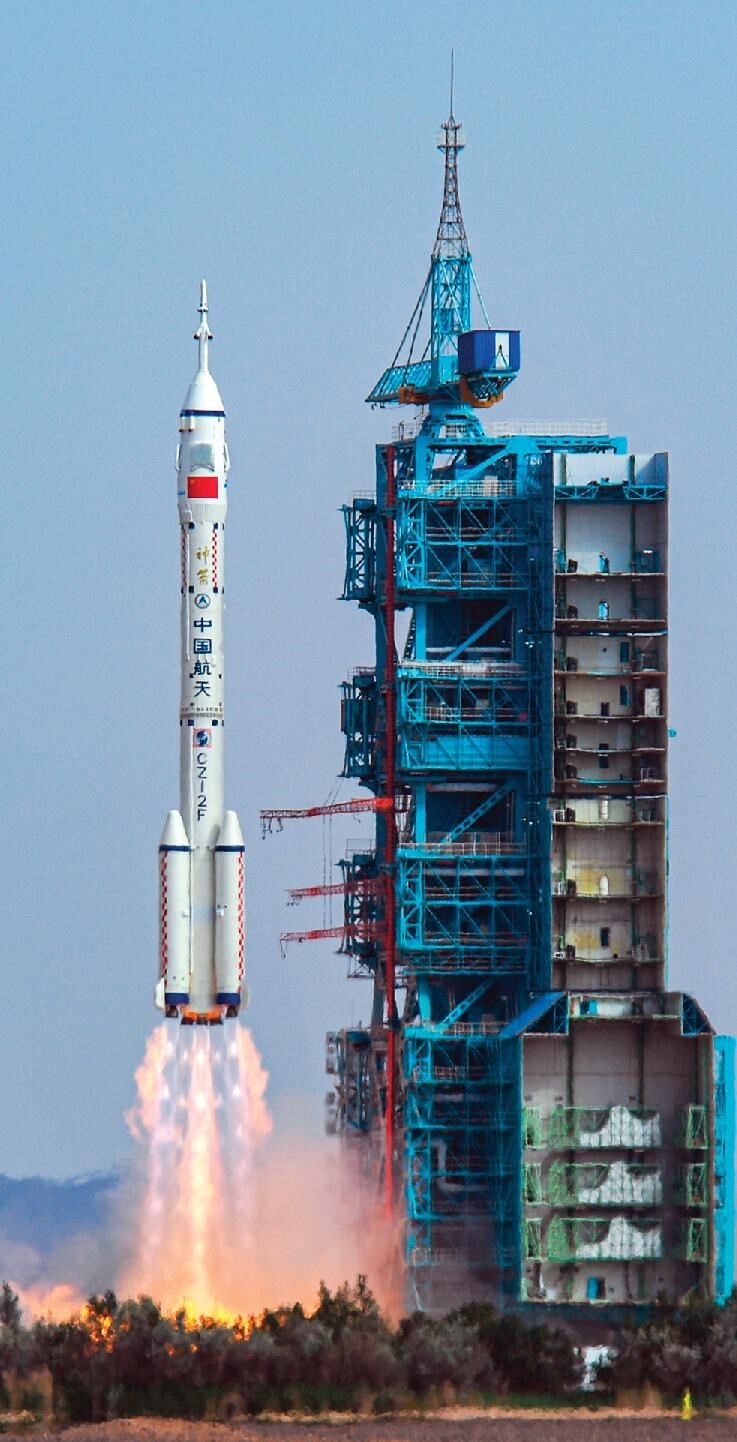
Shenzhou 16 spent 154 days in space, from 30 May to 31 October 2023. (Courtesy of China Manned Space.)

The Shenzhou 16 spacecraft took off on 30 May 2023 at Jiuquan Launch Center and docked with the Tianhe core module (Fig. 2). As the first mission crew during the application and development stage of the space station, the 3 astronauts remained in orbit for 154 days. They conducted one spacewalk, gave the fourth live lecture from the space station and transferred multiple cargo items to the exterior. These activities built the foundation of normal operations at the space station.

With close cooperation with ground crew, the space crew conducted a number of aerospace experiments in genetic engineering, aerospace medicine, biotechnology, space technology, materials science, aerodynamics and ecology. They made important progress in life sciences in space, micro-gravity physics and other areas. As far as manned space exploration is concerned, this mission represents a major step from construction to application, from investment to production.

## 3. New 2D transistor surpassing the limits of silicon-based technologies.

**Figure 3.**
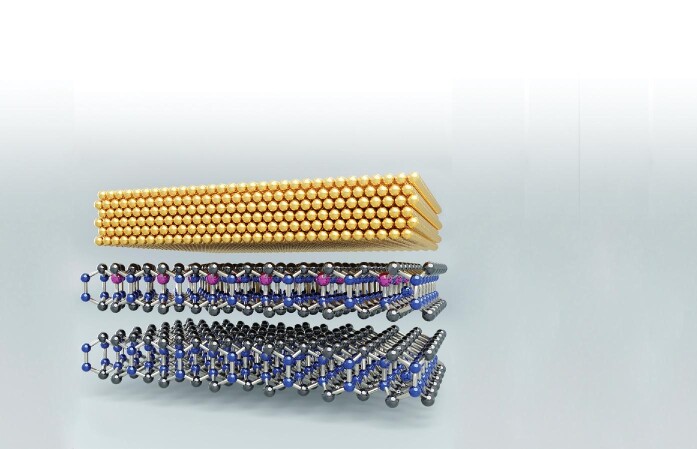
Molecular structure of the InSe 2D transistor with Y-doping (pink). (Courtesy of Profs. Lianmao Peng and Chenguang Qiu of Peking University.)

Computer chips are at the center of the information age, but their further development is constrained by traditional technology approaching its physical limit in terms of size and density. 2D semiconductors with atom-scale thickness theoretically have more potential, but they have not surpassed the industrial standards of commercial silicon technology. The research team led by Profs. Lianmao Peng and Chenguang Qiu of Peking University constructed 2D indium selenide transistors with 10 nm channel length based on an yttrium-doping-induced phase-transition theory [1] (Fig. 3). Using an atomic-scale precision doping method, they successfully overcame the major obstacle in 2D materials research that is the contact between metal and semiconductor. For the first time, they manufactured 2D transistors that exceeded the best performance of 10 nm silicon field effect transistors by reaching an operational voltage of 0.5 V and a room-temperature ballistic ratio in the saturation region of 83%. This type of transistor is currently the fastest and most energy-efficient in the world.

## 4. A newly discovered gene linking alkaline tolerance with improved crop productivity.

**Figure 4.**
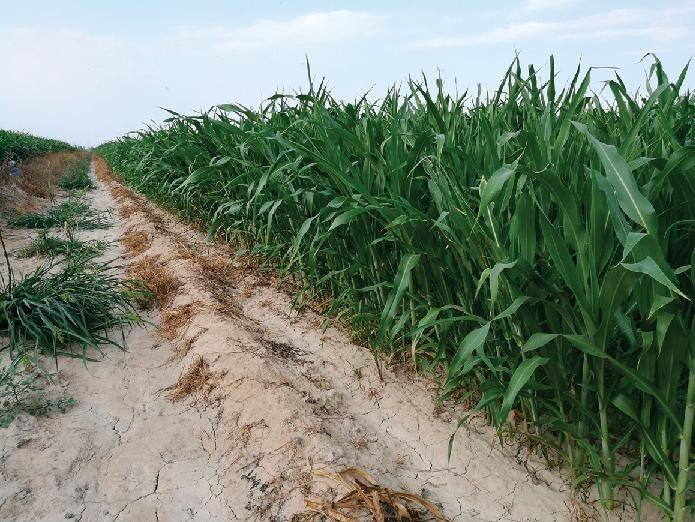
The major genetic locus (AT1) may significantly improve crop production. (Courtesy of IGDB, CAS.)

Approximately 100 million hectares of farm land in China are saline-alkali, making up nearly one-tenth of the worldwide total. However, salinization of farmable land continues to increase with climate change, water shortage and massive usage of fertilizers. The research team of Prof. Qi Xie of the Institute of Genetics and Developmental Biology (IGDB), CAS, and his collaborators, discovered that a major genetic locus, Alkaline Tolerance 1 (AT1), may significantly improve crop production of sorghum, rice, wheat, corn and millet in saline-alkali land [2] (Fig. 4). This discovery shows great promise in the utilization and optimization of saline-alkali land and will play a crucial role in supporting food security.

## 5. Climate change on Mars revealed in Tianwen-1 data

Mars bears the most resemblance to Earth in the solar system. Research on Martian climate change has received considerable attention, as the status and evolution of the Martian environment is regarded as the ‘future of Earth’. Dunes formed by wind have shaped Martian surface features, with earlier cementation to later erosion. These features record late-stage evolution and recent climate activities on Mars. However, due to the lack of localized and close-range scientific observations, we know little about the past activities of wind, sand and ancient climate.

**Figure 5.**
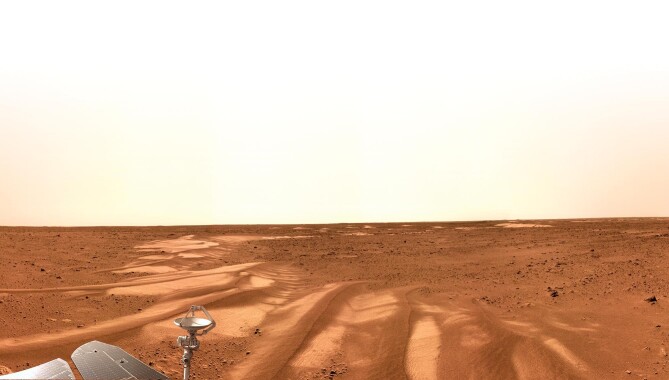
A panoramic photo taken by the Zhurong Mars rover. (Courtesy of NAO, CAS.)

The team that tackled this scientific problem included Chunlai Li of the National Astronomy Observatory, CAS, Zhengtang Guo of the Institute of Geology and Geophysics, CAS, and other researchers from the Institute of Tibetan Plateau Research, Brown University, and the Tianwen-1 Engineering team. Together, they focused on a southern region rich in geological features in Utopia Planitia, with a battery of scientific instruments on the Zhurong Mars rover including a high-resolution panoramic camera (Fig. 5), a navigational terrain camera, a multispectral camera, a meteorology monitor and a surface composition detector. By combining remote sensing with close-range measurements, these instruments acquired data such as dune morphology, surface structure and material composition. Later analysis provided wind direction, terrain evolution and other information about the rover's landing area. Evidence showed that the landing area may have experienced two major climate periods with distinct directions of wind [3]. This finding is consistent with ice records from mid-to-high latitude regions on Mars. A global change in the Martian climate occurred ∼400 000 years ago at the end of the last glacial age and may have been caused by a change of obliquity of Mars. This research enhances our understanding of ancient climates on Mars and provides a source of reference as we study climate change on Earth.

## 6. Drilling started at the first 10 000 m oil field in China.

**Figure 6.**
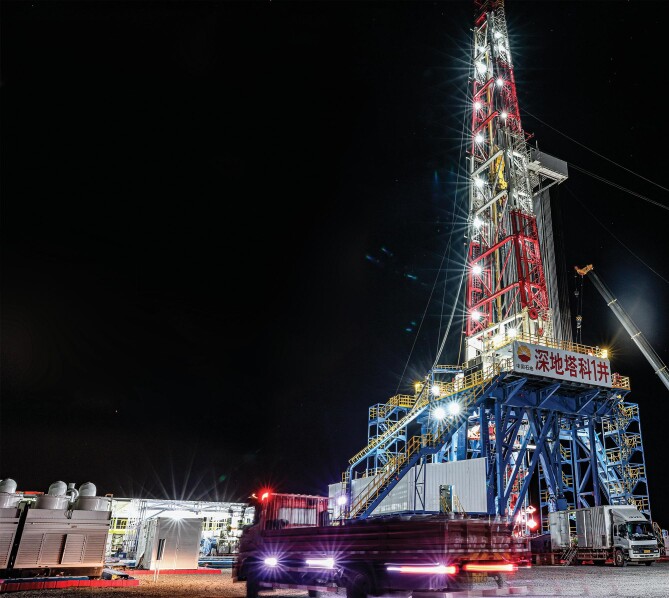
(Courtesy of China Petroleum and Natural Gas Co.)

Drilling started at Shendi Tako Well No. 1 in the Tarim oil field of China Petroleum and Natural Gas Co. on 30 May 2023 (Fig. 6). This drilling represents an advancement of geological science and engineering at the 10 000 m range and signifies a major breakthrough in the technologies of deep-Earth detection.

Shendi Tako Well No. 1 is located in Shaya county in Xinjiang, in close proximity to a region with an oil and gas reserve of 1 billion tons at 8000 m deep. This well is expected to be 11 100 m deep and the drilling is expected to take 457 days. The drilling plan will set the fastest drilling record for wells over 10 000 m deep. The drilling will be performed by the first 12 000 m driller in the world, developed domestically in China. This driller has a maximal weight load of 900 tons, compared to the 300–400 tons of a conventional driller. It can lift 150 adult elephants simultaneously. In order to complete the drilling successfully and on time, China Petroleum and Natural Gas Co. developed core technologies such as an automated intelligent control system and a super-heavy weigh chassis. This driller sets a foundation for more future explorations of science and engineering at the 10 000 m range.

## 7. A nickelate superconductor discovered for the first time at liquid nitrogen temperature.

**Figure 7.**
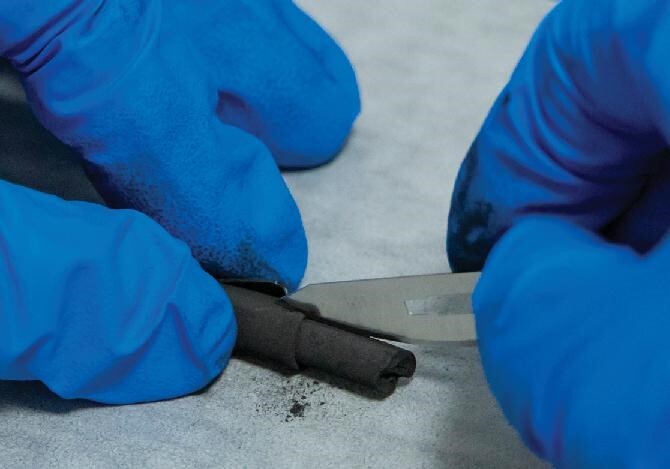
La3Ni2O7 is only the second non-conventional superconductor ever discovered at liquid nitrogen temperature. (Courtesy of Prof. Meng Wang's group at Sun Yat-Sen University.)

The research group of Prof. Meng Wang of Sun Yat-Sen University and their collaborators at Tsinghua University and South China University of Technology published their discovery in July 2023, which concerned the first nickelate superconductor at the liquid nitrogen temperature of 80 K under a pressure of 14 Gpa [4] (Fig. 7). As a major breakthrough in basic science, this material is the first all-new superconductor family discovered by Chinese scientists and only the second non-conventional superconductor ever discovered at liquid nitrogen temperature. Prior studies only indicated cuprate bulk materials with unconventional superconductivity above the liquid nitrogen boiling temperature of 77 K. This discovery of the nickelate crystal (La_3_Ni_2_O_7_) may advance our understanding of the mechanism of high-temperature superconductors and help us to design and predict more superconductors of this type. It may expand the applications of superconductivity in information science, manufacturing, power generation, biomedicine and transportation.

## 8. Evidence of nanohertz gravitational waves detected by the Five-hundred-meter-Aperture Spherical radio Telescope (FAST).

**Figure 8.**
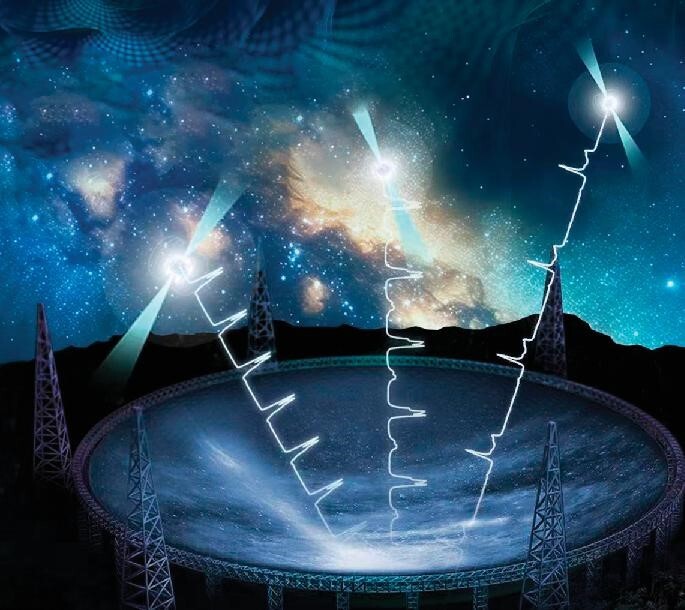
(Courtesy of the Chinese PTA group.)

The Chinese pulsar timing array (PTA) research group consisting of scientists from the National Astronomical Observatory (NAO) and other institutions detected crucial evidence of nanohertz gravitational waves using FAST, a world class achievement by Chinese scientists in the detection of nanohertz gravitational waves (Fig. 8). This result was published on 29 June 2023 in the domestic astronomy journal *Research in Astronomy and Astrophysics* [5].

Nanohertz gravitational wave detection has now become a focus of international competitions in physics and astronomy due to its challenging nature, because the low frequencies of nanohertz gravitational waves result in oscillation periods of several years and wavelengths of several lightyears. The only known method of detection is by long-term observation of nanosecond pulsars using large-scale radio telescopes. It is worth noting that other pulsar timing arrays from Europe, India, North America and Australia announced similar results at this time. As a member of the Chinese PTA team, Prof. Kejia Lee from Peking University and NAO emphasized the accuracy and reproducibility of this result, as four international teams have detected this evidence independently.

## 9. The world's first full-link and full-system space-based solar power station commencing ground verification.

**Figure 9.**
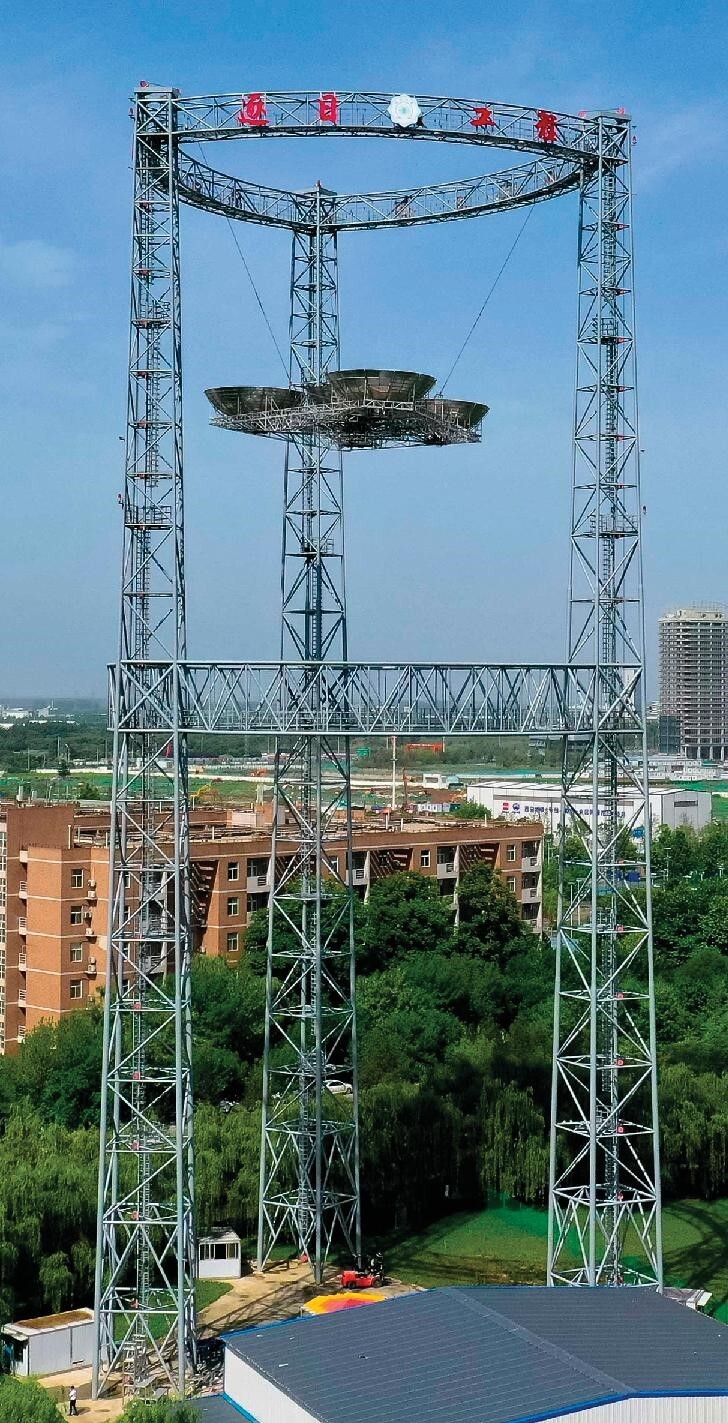
The technology of the Zhuri project transmits 2081 watts over 55 meters. (Courtesy of Prof. Baoyan Duan of Xidian University.)

A space-based solar power station (SSPS) is one of the proposals to solve the energy crisis and eventually achieve sustainable development. This technology consists of collecting solar power in space and transferring it to Earth's surface in the form of wireless microwaves. The research team of Prof. Baoyan Duan at Xidian University published their SSPS project on 30 November 2023 in the journal *Engineering*, the flagship journal of the CAE [6]. Their project, Zhuri, aims to be the ground verification system of the world's first full-link and full-system SSPS. The system has verified the technology for the high-power wireless transfer of 2081 watts over 55 meters (Fig. 9). The reception efficiency of the microwaves reached 87.3% with a DC-DC transfer efficiency of 15.05%. The power-to-mass ratio and other parameters of the system are also leading similar projects in the world.

The Zhuri project represents a breakthrough in wireless power transfer, with broad applications in space, such as for power grids, charging stations, satellite communication, computation and super-long-range remote sensing. On Earth, this technology can provide power for airships, drones, service stations in the ocean, remote areas and areas in disaster.

## 10. New insights made regarding the olfactory system and its molecular mechanism.

**Figure 10.**
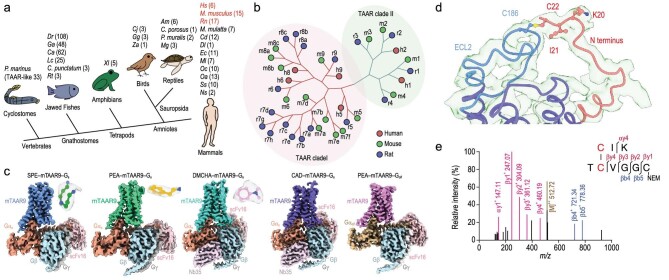
The overall structure of the mTAAR9–Gs and mTAAR9–Golf complexes. (Reproduced from ref. [7] with permission. Copyright 2023, Springer Nature.)

Most mammals, including humans, possess an olfactory system, or a sense of smell, to distinguish volatile and odorant molecules. Trillions of odorant molecules may be identified through an encoding mechanism of two families of receptors, the odorant receptors and the trace amine-associated receptors (TAARs). One member of the TAAR family of receptors in mice, mTAAR9, was studied by the research group of Profs. Jin-Peng Sun of Shandong University and Qian Li of Shanghai Jiaotong University, using cryo-electron microscopy (Fig. 10). They discovered that mTAAR9 molecular structures contain a specific ligand-binding pocket that is essential for recognizing amine odorants [7]. In addition, they identified shared structural motifs of TAAR family receptors to be activated by the same odorants. Collectively, this research uncovers the molecular mechanism of TAAR receptors and provides a theoretical foundation for olfactory coding and identification. It is of great significance in the development of new drugs targeting olfactory receptors.

## Author Biography

He Zhu (朱禾) is a science and news editor at the editorial office of NSR in Beijing, China.

## References

[1] Jiang JF, Xu L, Qiu CG et al. Nature 2023; 616: 193–200. 10.1038/s41586-023-05819-w

[2] Zhang HL, Yu FF, Xie P et al. Science 2023; 379: 1024.

[3] Liu JJ, Qin XG, Xin R et al. Nature 2023; 620: 303–9. 10.1038/s41586-023-06206-1 37407822 PMC10412455

[4] Sun HL, Huo MW, Hu XW et al. Nature 2023; 621: 493–8. 10.1038/s41586-023-06408-7 37437603

[5] Xu H, Chen SY, Guo YJ et al. Res Astron Astrophys 2023; 23: 075024. 10.1088/1674-4527/acdfa5

[6] Duan BY, Zhang YQ, Chen GD et al. Engineering 2023; 10.1016/j.eng.2023.11.007.

[7] Guo LL, Cheng J, Lian S et al. Nature 2023; 618: 193–200. 10.1038/s41586-023-06106-4 37225986

